# Impact of Guidelines Publication on Acute Bronchiolitis Management: 10-Year Experience from a Tertiary Care Center in Italy

**DOI:** 10.3390/microorganisms9112221

**Published:** 2021-10-26

**Authors:** Carlotta Biagi, Sara Scarpini, Camilla Paleari, Marianna Fabi, Arianna Dondi, Liliana Gabrielli, Monia Gennari, Marcello Lanari, Luca Pierantoni

**Affiliations:** 1 Pediatric Emergency Unit, Scientific Institute for Research and Healthcare (IRCCS), Sant’Orsola Hospital, 40138 Bologna, Italy; carlotta.biagi@aosp.bo.it (C.B.); marianna.fabi@aosp.bo.it (M.F.); arianna.dondi@aosp.bo.it (A.D.); monia.gennari@aosp.bo.it (M.G.); marcello.lanari@unibo.it (M.L.); luca.pierantoni@aosp.bo.it (L.P.); 2 Specialty School of Pediatrics, Alma Mater Studiorum, University of Bologna, 40126 Bologna, Italy; camilla.paleari@studio.unibo.it; 3 Microbiology Unit, Scientific Institute for Research and Healthcare (IRCCS), Sant’Orsola Hospital, 40138 Bologna, Italy; liliana.gabrielli@aosp.bo.it

**Keywords:** bronchiolitis, evidence-based guidelines, practice management, treatment, corticosteroids, beta 2 agonists, radiography, resource utilization

## Abstract

Bronchiolitis is the most common lower respiratory tract infection in infants. According to evidence-based guidelines, diagnosis is clinical, there is no need for routine use of laboratory or instrumental tests and therapy is primarily supportive, based on oxygen and adequate fluid supplementation. Nevertheless, unnecessary diagnostic tests and pharmacological treatments are still very common. The aim of this retrospective cohort study was to evaluate how the management of bronchiolitis has changed in the last ten years in a Tertiary Care Center in Italy, assessing adherence to national guidelines. Considering the publication of the Italian inter-society consensus document in 2014, we compared patients admitted in the prior four epidemic seasons with patients admitted in the latter six epidemic seasons. The comparison between the two groups showed a significant reduction in the prescription of systemic corticosteroids (58.9% vs. 41.8%, *p* < 0.001), nebulized epinephrine (73.8% vs. 38.3%, *p* < 0.001) and antibiotics (59.5% vs. 42.3%, *p* < 0.001), together with a drastic decrease in the use of chest X-ray (92.2% vs. 54.4%, *p* < 0.001). On the contrary, the use of inhaled salbutamol remained substantially stable over time (39.4% vs. 37.6%, *p* = 0.505). Despite the encouraging results, further efforts are needed to limit the prescription of ineffective therapies like antibiotics and inhaled salbutamol.

## 1. Introduction

Bronchiolitis is the most common lower respiratory tract infection (LRTI) in infants [1], with an increasing incidence [2] affecting approximately one child out of three during the first year of life, 2–3% of which end up being hospitalized [3]. Respiratory Syncytial Virus (RSV) is the main etiological agent [4,5,6], with infection typically occurring as recurrent seasonal epidemics in temperate climates, whereas in cold climates it is continuous throughout the year [3]. RSV is classified into two groups, A and B, based on antigenic variation and sequence analysis. This virus shows a complex circulation pattern, as numerous genotypes/subgroups within each group have been identified and co-circulation of genotypes within the same community is common [7,8]. Other viruses, such as rhinovirus, adenovirus, influenza, parainfluenza, metapneumovirus, and human coronavirus, are less frequently implicated [9,10]. Up to 30% of hospitalized infants with bronchiolitis have multiple respiratory virus co-infections [11]. 

The clinical picture of bronchiolitis is characterized by the initial appearance of rhinitis, which evolves after two to four days towards symptoms of LRTI including a hacking cough, tachypnea, widespread wheezing, and crackles at chest auscultation, dyspnea with increased respiratory efforts, and feeding difficulties [3].

Diagnosis is based on the history and physical examination, and there is no need for routine use of laboratory or instrumental tests [4]. Due to a lack of specific etiological treatment, therapy is primarily supportive, based on oxygen and adequate fluid supplementation.

To improve the standardization of the management of bronchiolitis, evidence-based guidelines (GL) have been developed worldwide [3,4,12], including the ones published between 2014 and 2015 by the National Institute for Health and Care Excellence (NICE) and by the American Academy of Pediatrics (AAP) [3]. An inter-society consensus document on treatment and prevention of bronchiolitis in newborns and infants has been published in October 2014 in Italy [13]. 

According to these guidelines, chest X-ray (CXR) should be reserved for severe bronchiolitis leading to intensive care unit (ICU) admission or for patients with signs of pulmonary complications (i.e., pneumothorax). Moreover, all these documents agree that supportive treatments (i.e., oxygen therapy and maintenance of hydration) are the cornerstone of bronchiolitis management, discouraging the use of pharmacological treatment. Salbutamol, epinephrine, systemic glucocorticoids, and antibiotics are not recommended [3,4,13]. Nevertheless, unnecessary diagnostic tests and pharmacological treatments are still very common, with more than 30% of infants hospitalized with bronchiolitis receiving no evidence-based therapies, with considerable inter-hospital variations [12,14]. 

This practice seems to depend on the age of young children affected and the potential severity of this infection. Concerning this awareness, physicians may feel forced to prescribe pharmacological therapies, either for personal reassurance or parental pressure [15]. Moreover, it seems that the GL features can influence the adherence to new recommendations [16]; deimplementation which includes discouragement of the prescription of ineffective tests and treatments for which there is no evidence, is particularly difficult when such practices are widely used [17].

The purpose of this study was to evaluate whether the Italian inter-society consensus document published in 2014 had a significant impact on the clinical and therapeutic management of bronchiolitis. To assess adherence to the Italian guidelines, patients with bronchiolitis hospitalized before and after 2014 were compared in terms of variation in the utilization of CXR and non-recommended pharmacotherapies.

## 2. Materials and Methods

### 2.1. Study Design

This was a retrospective cohort study conducted at the Pediatric Emergency Unit (PED) of S. Orsola University Hospital in Bologna, the largest tertiary pediatric referral center in the Emilia-Romagna region, Northern Italy. The PED is composed of a pediatric emergency room (ER) with an average of 24,000 visits per year of children aged 0–14 among the study period, a short-stay observation with six beds, and a ward with 28 beds.

We included all children admitted to the Pediatric Emergency ward for acute bronchiolitis between 1 October 2010 and 30 April 2020, considering 10 epidemic seasons. 

The primary endpoint of the study was to compare two groups, considering the publication of the Italian recommendations in 2014 as a reference: the first group (pre-GL group) included children hospitalized for bronchiolitis from October 2010 to April 2014, combining four epidemic seasons; the second group (post-GL group) comprised children admitted from October 2014 to April 2020, combining six epidemic seasons. Season 2020/21 (pandemic SARS-CoV period) has not been included in the analysis because no patients with bronchiolitis were hospitalized during this period [18]. Patients in the two groups were compared in terms of variation in the utilization of CXR and non-recommended pharmacotherapies.

Secondary endpoints were to compare clinical outcomes (incidence of complications, duration of oxygen administration, ICU admission, length of hospital stay) in the two study groups. 

### 2.2. Case Definition

“Acute bronchiolitis” was defined by the attending clinician on the basis of the interpretation of a constellation of characteristics findings including, but not limited, to: onset with rhinorrhea and/or upper respiratory tract infections; first episode of respiratory distress associated with crackles and/or wheezing, use of accessory muscles, low oxygen saturation levels, tachypnea, fever; exposure to persons presenting with upper respiratory tract viral infections; presentation during the epidemic season. According to the Italian recommendations for bronchiolitis, only infants less than 12 months at hospitalization were included [13].

Criteria for the definitions of “mild”, “moderate”, and “severe bronchiolitis” are shown in Table 1, as reported by the Italian inter-society consensus document [13].

“RSV bronchiolitis” was defined on the basis of the RSV detection on the nasopharyngeal aspirate.

### 2.3. Data Collection and Participants

Participant eligibility was defined through a hospital database search for diagnosis coded by the physicians at discharge according to the ICD (9th revision) (diagnosis codes 466.11 and 466.19). 

Data were extracted from scanned medical charts and electronic records and included the following information for each patient: basic demographic, epidemiological, patient history of co-morbidities and peri-natal characteristics (age, gender, breastfeeding, comorbidities, risk factors for bronchiolitis, time between the onset of symptoms and the hospital admission), vital signs at the arrival at the ER (heart and respiratory rate, body temperature, and oxygen saturation), clinical severity, laboratory (blood count, C-reactive protein, RSV test) and instrumental examinations (CXR), length of hospital stay, complications (bacterial co-infections like bronchopneumonia, acute otitis media, urinary tract infection, and sepsis; pneumothorax; pleural effusion). 

In addition, we collected data on treatment (antibiotic therapy, systemic corticosteroid, nebulized epinephrine and/or salbutamol, oxygen therapy, intravenous hydration), mode of administration of oxygen therapy (low-flow nasal cannula, Hood tent, high-flow nasal cannula (HFNC), noninvasive ventilation (NIV) including nasal continuous positive airway pressure ventilation (n-CPAP) or bilevel positive airway pressure ventilation (Bi-PAP), mechanical ventilation) and its duration in hours.

### 2.4. Inclusion and Exclusion Criteria

Inclusion criteria for the study were the admission to the Pediatric Emergency ward between 1 October 2010 and 30 April 2020, an attending physician’s diagnosis of acute bronchiolitis (ICD-9 466.11 and 466.19), and <1 year of age. Exclusion criteria were >1 year of age, and parent/guardian who refused collection or future use of data.

### 2.5. Diagnostic Tests

No specific diagnostic tests have been used in our study with the exception of a microbiological test for the diagnosis of RSV bronchiolitis. The aim of this test was cohorting patients with RSV to prevent hospital transmission, as contemplated by the Italian inter-society consensus document [13]. The microbiological diagnosis of RSV infection was performed by antigen detection from nasopharyngeal aspirates using a commercially direct immunofluorescence assay of cell smears, SimulFluor RespiratoryScreen (Light diagnostics TM, EMD Millipore Corporation). The assay sensitivity and specificity were found equal to 93.5% and 99.6%, respectively [19].

### 2.6. Statistical Analysis

Categorical variables are presented as numbers and relative percentages and they are compared by Pearson Chi-square test or Fisher exact test, as appropriate. Ordinal and continuous ordinal variables were tested for normal distribution by the Komolgorov-Smirnov test. Non-parametric variables are reported as median and relative interquartile range and compared by the Mann-Whitney test. Comparison between parametric variables, reported as mean and standard deviation, has been performed by Student’s t-test. All hypotheses were tested using two-tailed tests within a range of *p* < 0.05. All data analyses were performed using the Statistical Package for Social Sciences (SPSS) program, version 25.0 for Windows (SPSS, Chicago, IL, USA).

### 2.7. Ethics Statement

The study was conducted in accordance with the Declaration of Helsinki, and it was approved by the ethics committee of our institution (Ethics Committee Area Vasta Emilia Centro, AVEC, approval number 1062/2020/Oss/AOUbo).

## 3. Results

From October 2010 to April 2020, 1293, children under one year of age were admitted to our hospital for acute bronchiolitis. Forty-four of these patients were excluded from the study due to the absence of digitized medical records. The total number of patients enrolled was therefore 1249. 1201 (96.2%) patients came from the ER region and 4.4% of these spent a period in the short-stay observation before the admission to the ward, while the remaining 48 (3.8%) children have been hospitalized following an outpatient examination. 

Pre-GL and post-GL groups included 538 and 711 patients, respectively. 

The demographic, laboratory, and clinical characteristics of the study population are summarized in Table 2. 

In regards to the personal data of the whole sample, 717/1249 (57.4%) were male and the average age was 3.13 months (range 1.76–5.46). As shown in Table 1, there were no significant differences between the two groups, with the male predominance found in the total sample maintained in Pre-GL and post-GL groups. There was no statistically significant difference in the proportion of premature children (Pre-GL group: 16.4% vs. post-GL group: 15.9%, *p* = 0.831) while regarding the presence of other risk factors (chronic lung or congenital heart disease, immunodeficiency, severe neurological or muscular disease, airway malformation), they were significantly more frequent in the post-GL group compared to Pre-GL group (23.0% vs. 17.7%, *p* = 0.023). Infants breastfed in the Pre-GL group were significantly higher compared to the post-GL group (45% vs. 38.9%, *p* = 0.000), while patients in the post-GL group had feeding difficulties in a higher percentage (71.8 vs. 64.1, *p* = 0.004). 

Upon arrival at the hospital, the median time since the onset of symptoms was three days (DS 2–5) in the whole study population, without statistical difference between pre-GL and post-GL groups (*p* = 0.304). The median respiratory rate was higher in the post-GL group compared to the pre-GL group (55 vs. 50, *p* < 0.001), with no differences in oxygen saturation. Regarding clinical severity, 721 children had a mild form of bronchiolitis (57.8%), 489 a moderate form (39.1%), and 39 a severe form (3.1%), with no statistically significant differences between the two groups (*p* = 0.654).

Considering the entire study population, about 65.7% of children with bronchiolitis tested positive for RSV. The percentage of RSV-positive patients resulted significantly higher in the post-GL group compared to the pre-GL group (68.3% vs. 62.8%, *p* = 0.028). 

Analyzing blood tests results, we found a statistically significant increase in white blood cell count (WBC), lymphocytes count, and C-reactive protein (CRP) values in the post-GL group compared to the pre-GL group (Table 1). 

The evidence-based supportive therapies administered to the study population are shown in Table 3. 

The use of intravenous rehydration significantly increased, from 78.4% in the pre-GL group to 85.1% in the post-GL group (*p* = 0.002). Oxygen therapy was performed in 41.6% of children in the pre-GL group and in the 43.5% of the post-GL group, without differences in the number of patients treated (*p* = 0.519). In regards to the oxygenation modalities, we found a statistically significant increase in the use of a low-flow nasal cannula, from 61.2% in the pre-GL group up to 85.4% in the post-GL group (*p* < 0.001), and in the use of HFNC, rising from 1.7% in the pre-GL group to 10.4% in the post-GL group (*p* < 0.001). In contrast, there was a drastic decrease in the use of Hood tent, from 35.2% to 0.6%, respectively in the pre-GL group and post-GL group, basically due to the fact that this device fell into disuse. The percentage of patients receiving non-invasive ventilation (NIV) and mechanical ventilation (MV) was extremely low in both groups, with no statistically significant differences (Table 3).

### 3.1. Primary End-Point

Table 4 described the use of CXR and non-recommended pharmacotherapies in the different population groups. 

Regarding the utilization of CXR, we documented a substantial reduction in the number of CXR performed, with numbers almost halved in the post-GL Group (54.4%) compared to the pre-GL Group (92.2%) (*p* < 0.001). In particular, the number of CXR taken during hospitalization has decreased, season by season, from 96.7% in season 2010/11 to 36.7% in 2019/2020.

Our analysis showed a significant reduction in the administration of systemic corticosteroid from 58.9% to 41.8% (*p* < 0.001), antibiotic from 59.5% to 42.3% (*p* = 0.000) and nebulized epinephrine from 73.8% to 38.3% (*p* < 0.001) between pre-GL and post-GL group, respectively. On the other hand, the use of inhaled salbutamol remained substantially stable over time, with no significant differences between the pre-GL and post-GL groups (39.4% vs. 37.6%, *p* = 0.505). The rate of administration of salbutamol was higher in older infants in both study groups: more than 80% of infants over six months of age received salbutamol (88.6% in the pre-GL group, 81.9% in the post-GL group), compared to about 26% of younger infants 0–6 months old (26.2% in pre-GL group, 26.3% in post-GL group). In children who have obtained a clinical improvement thanks to nebulized salbutamol, the treatment was carried out throughout the duration of the whole hospitalization, with gradual discontinuation after discharge.

**Figure 1:**
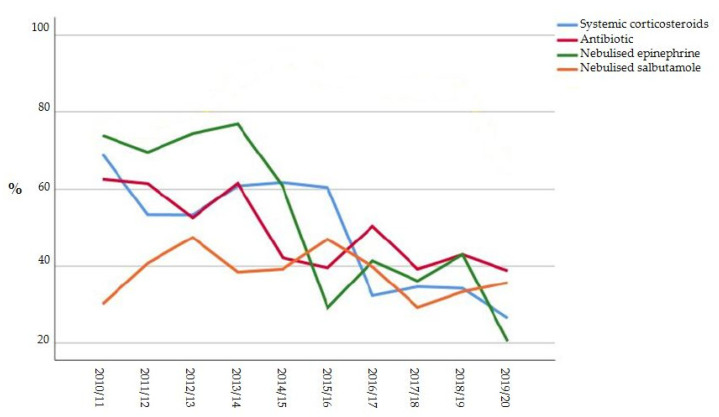
Variation in the use of pharmacologic treatments and CXR over time.

The variation in the use of pharmacologic treatments and CXR over time is reported in Figure 1.

### 3.2. Secondary End-Points

Secondary endpoints (incidence of complications, duration of oxygen administration, ICU admission, length of hospital stay) in the two study groups are reported in Table 5.

The most frequent complication associated with bronchiolitis turned out to be pneumonia: this was reported in 371 cases of the whole population (29.7%), of which 250 belonged to the pre-GL group (46.5%), and the remaining 121 to the post-GL group (17.0%), with a statistically significant reduction in the latter group (*p* < 0.001). The mean length of oxygen therapy did not differ in the two study groups (29.7 h in the pre-GL group vs. 33.7 h in post-GL groups, *p* = 0.505). Similarly, there was no difference in the percentage of infants requiring ICU admission (*p* = 0.217), while the mean length of hospital stay was higher in the pre-GL group compared to the post-GL group (5.5 days vs. 4.0, *p* < 0.001).

## 4. Discussion

The present study aimed to assess how the management of hospitalized children with acute bronchiolitis has changed in an Italian Tertiary Care Center after the publication of the national inter-society consensus document in 2014. 

To our knowledge, this is the first study describing the impact of bronchiolitis GL on non-evidence-based investigations and treatments in an Italian hospital setting. Recently Barbieri et al. investigated the prescriptions for bronchiolitis in the Italian pediatric primary care setting from December 2012 to December 2018, analyzing data from the Pedianet database [20]. They reported few changes after the GL publications, with 57% of patients still receiving unnecessary medicine. In particular, the antibiotic prescriptions rate remained almost stable, falling only from 33.5% to 31.6% of cases [20]. These results are even more alarming in the primary care context, where children usually present with mild symptoms. 

According to the literature, in our population, there was a predominance of males with an average age of three months [1,21,22]. Overall, the study population was homogeneous and no differences in sex, age, laboratory data, or bronchiolitis severity emerged between pre-GL and post-GL groups. This allowed minimizing bias at the time of comparison between the two study groups regarding the therapeutic management of bronchiolitis. The only meaningful difference between the two groups was a higher incidence of RSV infection in the post-GL group compared to the pre-GL group (68.3% vs. 62.8%, *p* = 0.028), together with a higher prevalence of risk factors for more severe disease (23% vs. 18%, *p* = 0.023). These percentages were coherent with the literature: RSV is detected in 41–83% of patients, and up to 34% of high-risk patients are reported [1,21,22]. 

According to national guidelines, laboratory investigations are not indicated for the routine workup of infants with bronchiolitis. However, regarding our study population, blood tests were carried out in all patients as a routine procedure for children admitted to the ward, using the same phlebotomy performed to place venous access necessary for rehydration. Due to this routine procedure, we cannot provide data about the impact of the guidelines on laboratory investigations.

Moreover, the AAP [4] and NICE GL [3] do not recommend respiratory viral testing in children with bronchiolitis. However, an etiological diagnosis may be useful in hospitalized patients to minimize the risk of nosocomial infections, by cohorting infants with the same causative virus [13]. According to this, the policy choice of our institution is to test every hospitalized child with bronchiolitis. 

Regarding CXR, it’s well known that it frequently leads to unnecessary use of antibiotics in children with bronchiolitis because of the similar radiographic appearance of infiltrating and atelectasis [23,24]. For this reason, CXR should be reserved for severe cases in which signs of pulmonary complications are present or where the severity of respiratory effort leads to ICU admission. Nevertheless, it continues to be performed frequently in many countries [14,25,26]. Our study clearly showed a marked reduction in CXR use after 2014, practically halved from 92.2% in the pre-GL group to 54.4% in the post-GL group (*p* < 0.001), reaching 36.7% in the 2019/2020 epidemic season. This encouraging data was also found in other works which described changes brought by the publication of AAP GL in 2006 [27,28] and more recently in the study published by House et al. [29]. This latter study is one of the few works that consider a significant time period after the update of the AAP GL in 2014. House and colleagues analyzed changes in the use of non-recommended services for bronchiolitis in US children’s hospitals over time, from November 2006 to December 2019. They found a significant decrease in the use of CXR in the inpatient group, passing from 78.4% in 2006 to 51.1% in 2014, and reaching 37.3% in 2019 [29]. The introduction of lung ultrasounds in the last decade as an emerging tool for diagnosing pneumonia may have contributed to this reduction, limiting exposure to ionizing radiation in the pediatric population [30,31,32,33]. 

In regards to treatment, the cornerstones of bronchiolitis therapeutic management are represented by oxygen therapy and fluid supplementation. GL suggests that rehydration may be administered intravenously or by nasogastric tubes [13]; in our department, the intravenous route is preferred over nasogastric tube for the consolidated practice of place venous access with the same phlebotomy performed for obtaining blood samples at admission. In our population study, intravenous hydration was administered in a higher percentage of patients in the post-GL group compared to the pre-GL group (85.1% vs. 78.4%, *p* = 0.002). This significant increase may be partly justified by the higher number of patients with feeding difficulties in the post-GL group (78.1% vs. 64.1%, *p* = 0.004).

Regarding oxygen supplementation, no significant variations in its use emerged between the two study groups, involving almost one child in two (42.7%) of the total sample. The results obtained in this study are in line with the clinical picture severity of the study population, evenly distributed in favor of mild-moderate forms in both study groups. According to this, Cheung et al. suggest there may be a recent trend toward hospitalizing children with milder bronchiolitis [34]. 

In regards to the respiratory support modes, low-flow nasal cannulas were the most commonly used in both groups, representing the first line respiratory support in cases of mild respiratory distress. In cases of more severe clinical conditions, the two groups have been treated with different devices: Hood tent has gradually fallen into disuse since the 2011/12 epidemic season, meanwhile, HFNC has been introduced with success in the pediatric field. HFNC is considered an effective rescue strategy in reducing the need to enhance respiratory support in children with bronchiolitis [35,36], offering the possibility of delivering heated, humidified oxygen with a flow of 1–2 L/kg/min [13]. In this regard, there was a large increase in their use in the post-GL group, rising from 1.7% in the pre-GL group to 10.4% in the post-GL group (*p* < 0.001), which has also been reported in the UK [37]. The low rate of access to ICU, especially in the last epidemic season, where admissions of patients with risk factors and complications of bronchiolitis have increased, can be interpreted considering the evidence available on the use of HFNC.

Regarding non-evidence-based treatments, the administration of nebulized salbutamol did not show a significant reduction after 2014 in our study population, contrary to what is described by House et al. [20], who identified the drastic reduction of beta 2 agonist use as the main change after the AAP GL publication in 2014. This may be related to the fact that the Italian inter-society consensus document, in contrast to the AAP of 2014, still allow a therapeutic attempt with salbutamol aerosol in those patients with a prevalence of wheezing to chest auscultation, family history of allergy, asthma, and/or atopy, mostly older than six months; more prolonged treatment can follow only in cases of proven clinical improvement after the salbutamol trial [13]. We documented indeed a higher rate of administration of salbutamol in infants older than six months in both study groups: more than 80% of infants over six months of age received salbutamol, compared to about 26% of younger infants. 

Regarding the use of systemic corticosteroids and nebulized epinephrine, in accordance with literature data [29,38,39], we found a significant reduction over time: systemic corticosteroid was administered in 58.9% of patients in the pre-GL group and in only 41.8% of children in the post-GL group (*p* < 0.001), and nebulized epinephrine fell from 73.8% to 38.3% (*p* < 0.001).

In line with national recommendations, we found a significant reduction in the use of antibiotics, decreasing from 59.5% in the pre-GL group to 42.3% in the post-GL group (*p* < 0.001). However, this result is still very high compared to what was expected. In fact, under certain conditions, such as documented or highly suspected bacterial over-infections and as a preventive treatment in children transferred to ICU and subjected to intubation and mechanical ventilation, it is appropriate to administer antibiotic therapy during bronchiolitis [40], but these situations are very rare. Zipursky et al. analyzed international patterns of antibiotic use: in their study 180 out of 2359 (7.6%) infants hospitalized for bronchiolitis received antibiotics, ranging from 3.5% in the United Kingdom and Ireland to 11.1% in the United States; this percentage rises to 21.6% for patients admitted to ICU (6). Thus, considerable differences in treatment between the various countries seem to emerge. In a study carried out in Canada to analyze the use of antibiotics in bronchiolitis before and after the national GL publication, it was found that the rates of antibiotic initiation were almost equal over time (about 44%). However, more antibiotics were discontinued during hospitalization and fewer patients were discharged with antibiotics in the post-GL group, illustrating that antibiotic prescribing practices can change [41]. Analyzing the impact of national GL in children hospitalized with bronchiolitis in public Portuguese hospitals, Fontoura-Matias et al. showed only a modest improvement in management, including antibiotic prescription, with significant variation between regions [42]. Regarding Italy, as we mentioned before, Barbieri et al. analyzed antibiotic prescriptions rate in the primary care context, which still stands at around 37% [20]. The high rate of antibiotic use in our country is therefore confirmed by the present study.

To confirm the improvements in the clinical management of bronchiolitis following the publication of the Italian GL in 2014, in our study we found a significant reduction in the mean length of hospital stay, with 5.5 days in the pre-GL group and 4.0 days in the post-GL group (*p* < 0.001). Despite the period of hospitalization having remained slightly longer than the international average (3–4 days), in our opinion this reduction could highlight the positive impact of a “less is more” management. Moreover, no differences emerged concerning the average length of oxygen therapy in the two study groups and the percentage of infants requiring ICU admission [43]. 

This study has some limitations. First of all, this is a single-center study, thus our results may not be fully representative of the management of infants with bronchiolitis in Italy. Moreover, given its retrospective design, causality cannot be ascertained. Finally, it was necessary to remove 44 patients from the total sample due to the lack of availability of digital medical records. In any case, it is assumed that this did not affect the final result, being a limited number compared to the total number of children involved in the analysis. In addition to the large sample, as a further strength point, there is the long observation period, which included ten epidemic seasons, thanks to which it was possible to create two homogeneous comparison groups.

## 5. Conclusions

In this study, we found a significant reduction in the prescription of systemic corticosteroids, nebulized epinephrine, and antibiotics after the publication of the Italian inter-society consensus document in 2014, and a drastic decrease in the use of CXR. Despite the encouraging results obtained from the observation of 10 epidemic seasons, it is clear that further efforts are needed to limit the prescription of ineffective therapies, particularly antibiotics and inhalatory salbutamol. Our results highlight the need for the development of quality initiatives to optimize the management of bronchiolitis, including physicians’ education and development of family-friendly material about non-evidence-based therapies in bronchiolitis, to reach clinicians reassurance and avoid parental pressure.

## Figures and Tables

**Table 1:** Bronchiolitis severity score [13].

	Mild	Moderate	Severe
Respiratory rate	Normal to slightly increased	Increased	Markedly increased compared to normal values
Respiratory effort	Mild chest wall retraction	Tracheal tug Nasal Flare Moderate chest wall retraction	Marked chest wall retraction Nasal flare Grunting
Oxygen saturations	No supplemental oxygen requirement, O_2_ saturations > 95%	Saturations 90–95%	Saturations < 90%, may not be corrected by O_2_
Feeding	Normal to slightly decreased	50–75% of normal feeds	<50% of feeds, unable to feed
Apnoea	Nil	May have brief episodes	May have increasing episodes

**Table 2:** Demographic, laboratory and clinical data of the study population.

	Whole Population (*n* = 1249)	Group 1 (*n* = 538)	Group 2 (*n* = 711)	*p*-Value
Age, median (IQR), months	3.13 (1.76–5.46)	3.00 (1.50–5.84)	3.13 (1.83–5.34)	0.093 *
Age under 6 months, n (%)	990 (79.3)	424 (78.8)	566 (79.6)	0.695 °
Sex, n (%), male	717 (57.4)	309 (57.4)	408 (57.4)	0.986 °
Prematurity, n (%)	201 (16.1)	88 (16.4)	113 (15.9)	0.831 °
Other risk factors ^$^, n (%)	258 (20.6)	95 (17.7)	163 (23.0)	**0.023 °**
Lactation, n (%):				**<0.001 °**
Breastfeeding	444 (35.5)	240 (45)	204 (28.9)	
Mixed Lactation	577 (46.2)	203 (38.1)	374 (52.9)	
Artificial feeding	219 (17.3)	90 (16.9)	129 (18.2)	
Feeding difficulties, n (%)	854 (68.4)	345 (64.1)	509 (71.8)	**0.004 °**
Time between the onset of symptoms and the hospital admission, median (IQR), days	3 (2–5)	3 (2–5)	3 (2–5)	0.304 *
Respiratory rate, median (IQR)	55 (45–60)	50 (45–60)	55 (45–60)	<0.001 *
Oxygen saturations, median (SD)	97 (95–98)	97 (95–98)	95 (95–98)	0.201 *
Bronchiolitis severity ^+^, n (%):				0.654 °
Mild	721 (57.8)	313 (58.2)	408 (57.3)	
Moderate	489 (39.1)	212 (39.4)	277 (38.9)	
Severe	39 (3.1)	13 (2.4)	26 (3.6)	
RSV detection, n (%)	812 (65)	331 (62.3%)	481 (68.3%)	**0.028 °**
WBC count, median (IQR), mmc:	10,940 (8.425–14,090)	10,215 (7875–13,227.5)	11,280 (8707.5–14,910)	<0.001 *
Neutrophil count, median (IQR), %	37.4 (25.4–49.05)	36.6 (25.35–48.6)	37.6 (25.4–49.55)	0.334 *
Lymphocytes count, median (IQR), %	48.5 (37.4–59.7)	47.5 (36.4–57.95)	49.1 (38.1–60.7)	0.028 *
CRP, mean (SD), mg/dL	0.48 (0.11–1.55)	0.31 (0.07–1.23)	0.67 (0.17–1.71)	<0.001 *

Significant differences for *p*-values are indicated in bold; * Mann-Whitney test *p*-values, ° Pearson Chi-square test (or Fisher exact test, as appropriate) *p*-values. Group 1: pre-GL group, included children hospitalized for bronchiolitis from October 2010 to April 2014; Group 2: post-GL group, included children hospitalized for bronchiolitis from October 2014 to April 2020. ^$^ Other risk factors include: previous history of chronic lung or congenital heart disease, immunodeficiency, severe neurological or muscular disease, airway malformation. ^+^ according to the Italian inter-society consensus document on bronchiolitis (13). CRP: c-reactive protein; IQR: Interquartile range; RSV: Respiratory Syncytial Virus; SD: standard deviation; WBC: White Blood Cells.

**Table 3:** Use of supportive therapy in the different population groups.

	Group 1 (*n* = 538)	Group 2 (*n*= 711)	*p*-Value
Intravenous hydration, n (%)	422 (78.4%)	605 (85.1%)	0.002 °
Oxygen therapy, n (%)	224 (41.6%)	309 (43.5%)	0.519 °
Time of oxygen therapy, hour			0.285 *
Median (IQR)	0 (0–48)	0 (0–48)	
Mean (SD)	29.70 (58.52)	33.68 (60.57)	
Type of Oxygen therapy			**<0.001 °**
NC, n (%)	137 (61.2%)	264 (85.4%)	
HT, n (%)	79 (35.2%)	2 (0.6%)	
HFNC, n (%)	4 (1.7%)	32 (10.4%)	
n-CPAP/NIV, n (%)	2 (0.9%)	6 (1.9%)	
MV, n (%)	2 (0.9%)	5 (1.6%)	

Significant differences for *p*-values are indicated in bold; * Mann-Whitney test *p*-values, ° Pearson Chi-square test (or Fisher exact test, as appropriate) *p*-values. Group 1: pre-GL group, included children hospitalized for bronchiolitis from October 2010 to April 2014; Group 2: post-GL group, included children hospitalized for bronchiolitis from October 2014 to April 2020. HFNC: high-flow nasal cannula; HT: Hood tent; IQR: Interquartile range; MV: mechanical ventilation; n-CPAP: nasal continuous positive airway pressure ventilation; NC: nasal cannula; NIV: non-invasive ventilation; SD: standard deviation.

**Table 4:** Use of CXR and non-recommended pharmacotherapies in the different groups.

	Group 1 (*n* = 538)	Group 2 (*n*= 711)	*p*-Value
Systemic corticosteroid, n (%)	217 (58.9%)	297 (41.8%)	**<0.001**
Antibiotic, n (%)	320 (59.5%)	301 (42.3%)	**<0.001**
Nebulized epinephrine, n (%)	397 (73.8%)	272 (38.3%)	**<0.001**
Nebulized salbutamol, n (%)	212 (39.4%)	267 (37.6%)	0.505
Chest X-ray, n (%)	496 (92.2%)	387 (54.4%)	**<0.001**

Significant differences for *p*-values are indicated in bold; the statistical tests used to derive *p*-values are the Pearson Chi-square test or Fisher exact test, as appropriate. Group 1: pre-GL group, included children hospitalized for bronchiolitis from October 2010 to April 2014; Group 2: post-GL group, included children hospitalized for bronchiolitis from October 2014 to April 2020.

**Table 5:** Secondary endpoints in the different population groups.

	Group 1 (*n* = 538)	Group 2 (*n* = 711)	*p*-Value
Concomitant pneumonia, n (%)	250 (46.5)	121 (17.0)	<0.001 *
Other complications, n (%)	2 (0.37)	12 (1.68)	0.43 °
		
• Sepsis Urinary tract infection	1 (0.18)	2 (0.28)	
• Acute otitis media	1 (0.18)	1 (0.14)	
• Pneumothorax	0 (0)	5 (0.70)	
• Pleural effusion	0 (0)	3 (0.42)	
Time of oxygen therapy, hour			0.285 *
Median (IQR)	0 (0–48)	0 (0–48)	
Mean (SD)	29.70 (58.52)	33.68 (60.57)	
Length of hospital stay, mean (SD), days	5.5 (4–8)	4.0 (3–6)	<0.001 *
ICU admission, n (%)	7 (1.3)	16 (2.3%)	0.217 °

Significant differences for *p*-values are indicated in bold; * Mann-Whitney test *p*-values, ° Pearson Chi-square test (or Fisher exact test, as appropriate) *p*-values. Group 1: pre-GL group, included children hospitalized for bronchiolitis from October 2010 to April 2014; Group 2: post-GL group, included children hospitalized for bronchiolitis from October 2014 to April 2020. IQR: Interquartile range; SD: standard deviation.

## Data Availability

The data presented in this study are available on request from the corresponding author. The data are not publicly available due to reasons concerning privacy.
